# Supplementary material for: New estimates of flood exposure in developing countries using high-resolution population data
Source: Nat Commun. 2019 Apr 18;10:1814. doi: 10.1038/s41467-019-09282-y (PMC6472407; doi:10.1038/s41467-019-09282-y)
Supplement: Supplementary file 1 — Supplementary Information [file 41467_2019_9282_MOESM1_ESM.pdf]

Supplementary Information

New estimates of flood exposure in developing countries using high-resolution population data, Smith et.al.

Supplementary Figures

Title: Example of HRSL data in Malawi.

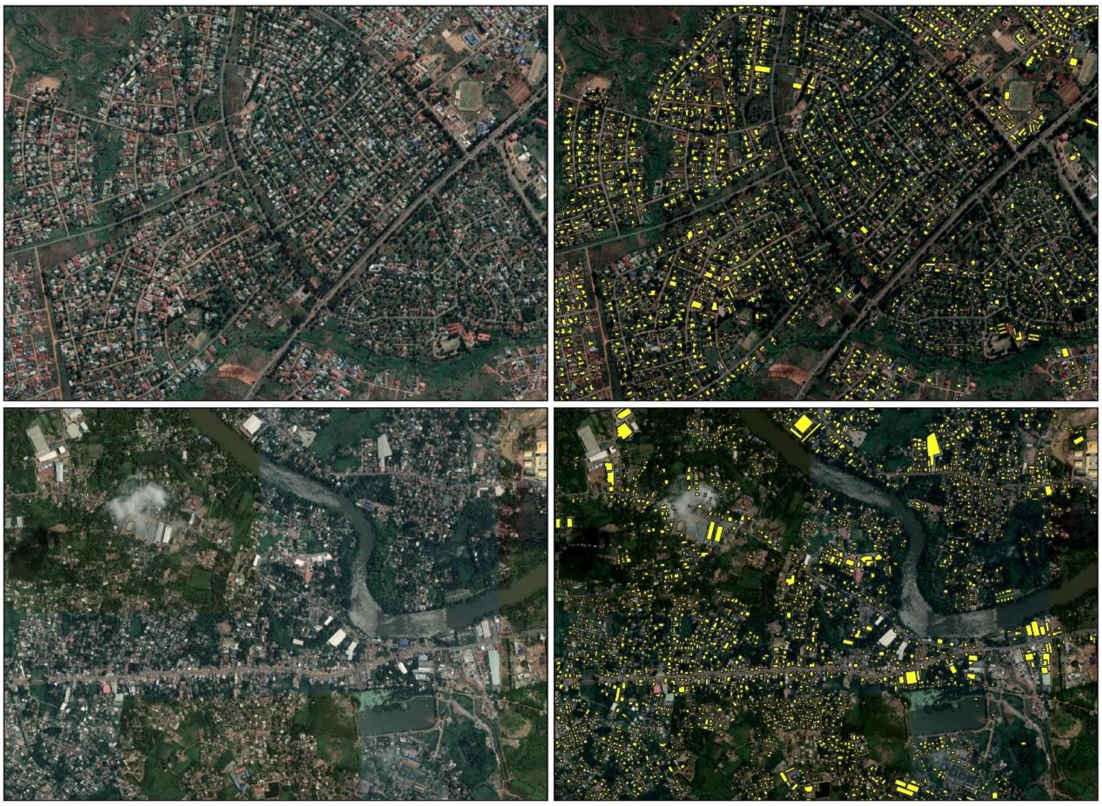

Supplementary Figure 1: Google Earth satellite imagery from Lilongwe, Malawi (top left) and Colombo, Sri Lanka (bottom left). Images of in the right show the corresponding OSM residential building data in yellow.

Supplementary Tables

Supplementary Table 1: A non-exhaustive summary of recent large-scale flood risk analyses and the population datasets used.

Model/ study name	Hydrology component	Flow routing compone nt	Inundation data resolution after downscaling	Population dataset	Population base data	Population downscaling method and final resolution	Population data resolution
GLOFRIS Ward et al.[1]	PCR-GLOBWB (0.5 degree) driven by EU-WATCH reanalysis 1960–1999	Kinematic wave **0.5 deg**	30 arc sec **~900 m**	LandScan™ Bhaduri et al.[2]	2010 population estimates for sub-national scale administrative units obtained from the US Bureau of Census for	Dasymetric downscaling approach using spatial data and imagery to disaggregate census data to a regular grid 30 arc sec **~900 m**	30 arc sec **~900 m**
CaMa-UT Hirabayashi et al. [3]	MATSIRO-GW (1 degree)-driven by JRA-25 Reanalysis 1979-2010 +GPCP rain gauge correction	Inertial wave **0.25 deg**	18 arc sec **~540 m**	Gridded Population of the World (GPW) version 3 CIESIN and CIAT[4]	Census data from 300,000 national and sub-national administrative units	Proportional allocation gridding algorithm to downscale from administration units to a regular grid. 2.5 arc minutes **~4500 m**	2.5 arc minutes **~4500 m**
CIMA-UNEP (GAR, [5])	Regional FFA from global gauge data + ECEarth bias corrected	Manning's equation at multiple points	3 arc sec **~90 m**	LandScan™ Bhaduri et al. [2]	2010 population estimates for sub-national scale administrative units obtained from the US Bureau of Census for	Dasymetric downscaling approach using spatial data and imagery to disaggregate census data to a regular grid 30 arc sec **~900 m**	Data aggregated to **1x1km** within 10km of a coastline, **5x5km** elsewhere
GLOFRIS, CaMA-UT, CIMA-UNEP, Fathom-Global90 (formerly known as SSBN), JRC, ECMWF Trigg et al. [6]	Various	Various	3-30 arc sec **~90-900 m**	WorldPop Stevens et al. [7]	2010 population from census data for sub-national scale administrative unit, with mapping of population from imagery data where census information are considered unreliable	Dasymetric downscaling based on land use and other data to disaggregate census data to a regular grid **100 m**	Data aggregated to 30 arc sec resolution to match coarsest model output **~900 m**
CaMa-UT Kinoshita et al. [8]	MATSIRO-GW (1 degree)-driven by JRA-25 Reanalysis 1979-2010 +GPCP rain gauge correction	Inertial wave **0.25 deg**	18 arc sec **~540 m**	History Database of the Global Environme nt (HYDE) version 3.1 for the year 2005 Goldewijk et al. (2010)[9]	Various national and sub-national historical population sources, see Goldewijk et al.[9] for details	Downscaling based on land use and Landscan™ population data 5 arc minutes **~10 km**	5 arc minutes **~10 km**
Fathom-US Wing et al [21]	Regional FFA from global gauge data	Inertial wave 1 arc second **~30 m**	No downscaling 1 arc sec **~30 m**	US Environme-ntal Protection Agency (EPA) EnviroAtlas Pickard et al. [10]	2010 US census block population counts	Dasymetric downscaling approach using land use and slope	1 arc sec **~30m**

Supplementary Table 2: Contingency table of possible cell descriptors in binary building classification.

	Building in OSM data	No building in OSM data
Building in Population data	P_1OSM_1	P_1OSM_0
No building in Population data	P_0OSM_1	P_0OSM_0

Supplementary Table 3: Hit rates (HR) for OSM buildings vs Population data.

**HR**	**HRSL**	**WP**	**LS**
**Haiti**	0.89	1.00	0.95
**Malawi**	0.96	0.99	1.00
**Sri Lanka**	1.00	1.00	1.00
**Phillipines**	0.96	0.98	0.68

Supplementary Table 4: False positive ratio (FP).

**FP**	**HRSL**	**WP**	**LS**
**Haiti**	0.09	0.90	0.90
**Malawi**	0.13	0.91	0.91
**Sri Lanka**	0.09	0.62	0.62
**Phillipines**	0.03	0.82	0.85

Supplementary Table 5: Critical Success index (CSI).

**CSI**	**HRSL**	**WP**	**LS**
**Haiti**	0.82	0.10	0.10
**Malawi**	0.84	0.09	0.09
**Sri Lanka**	0.91	0.38	0.38
**Phillipines**	0.94	0.18	0.14

Supplementary Discussion

Table 1 provides a summary of approaches taken to date in flood exposure analysis and highlights the varying spatial resolution and methods of both the physical models and population data used. The table highlights that many current gridded global population density datasets are of relatively coarse spatial resolution (30 arc seconds to 5 arc minutes, or ~900m to ~10 km at the equator). The only exception to this is the ~3 arc second WorldPop dataset

used by Trigg *et al*. (2016) [6]: the 1 arc second spatial resolution US Environmental Protection Agency EnviroAtlas data used by Wing *et al*. (2018) [11] are only available for the continental US. This coarse resolution is problematic as flooding is a highly localised phenomenon, with marked spatial variability at 25-100 m scales on rural floodplains[12] and at <10 m scale in urban areas [13].

HRSL Validation

To assess the accuracy of the new HRSL data, a building level validation exercise was undertaken whereby the HRSL along with the WorldPop and LandScan™ datasets were evaluated. Validation exercises of building level population data have been attempted previously, however these validation exercises are limited to areas in which bespoke detailed building level population data has been collected [14]. Very few such data sets have ever been collected, and to the best of our knowledge not at all in the developing world countries we consider here. Census data sets do exist for the countries we consider; however these are always aggregated to larger enumeration areas for privacy reasons. For these reasons, the direct comparison of the HRSL population data to existing building level populations is precluded by the lack of available validation data. However, at an aggregated level, the HRSL data produces similar values to the other population datasets used in this study. Indeed the population dataset that is disaggregated to form the HRSL, the Gridded Population of the World (GPW) v4, has been extensively evaluated in the literature [15–18]. This should perhaps not be surprising as all global gridded population data sets are largely derived from the same aggregated national census data.

Although the comparison of like-for-like building level population data in any of the HRSL countries is currently impossible, a benchmarking exercise defining how well each of the population datasets represent individual buildings was undertaken. The validation data used here were taken from the OpenStreetMap (OSM) project. OSM aims to create a free-to-use map of the world, including individual buildings, and has been shown to represent geo-spatial

information with average horizontal errors of ~6m[19]. For its application here, a visual inspection of OSM data was undertaken to identify areas in which the representation of buildings appeared accurate and comprehensive; this visual inspection compared OSM data against available remotely sensed imagery. Upon completing this inspection four regions were identified as being suitable areas for the provision of building benchmark data (Figure 1). These areas were Lilongwe – Malawi, Colombo – Sri Lanka, Manila – Philippines and Les Cayes – Haiti. These areas provided a total of ~74,000 building with which to benchmark the population data. To enable a direct comparison of OSM building data against the gridded HRSL, WorldPop and LandScan™ data, the OSM building shapefile data was transformed onto a 1 arc-second resolution grid.

For the sake of comparison here, each population data was transformed into a binary data-layer, whereby all non-zero cells were set to 1 and all zero cells were set to 0. These values were then assumed to correspond to building and no-building cells respectively. Three basic measures of fit were used to identify how well each population dataset represented the OSM building data, with each performance metric analysing the relative number of pixels conforming to the states outlined in the contingency table (Table 2). To account for uncertainty in the benchmark OSM data, and its transformation into a gridded dataset, the incidence of finding a building or not is measured within a distance of 1 arc second (30m) of the pixel being interrogated.

The first performance metric used is a simple hit rate (HR) which defines the proportion of building cells in the OSM data that are also represented in the population dataset being tested. HR can range from 0 (none of the OSM buildings were represented in the population data) to 1 (all the OSM buildings were represented in the population data).

$$HR = \frac{P_1\, OSM_1}{P_1\, OSM_0 + P_0\, OSM_1}$$

The second performance metric used was a false positive ratio (FP). In contrast to the HR metric, which identifies the tendency of the population data to under-predict benchmark (OSM) data, the FP metric identifies whether the population data tends to overestimate the presence of buildings in the OSM data. The FP metric can range from 0 (no false positives), to 1 (all false positives).

$$FP = \frac{P_1\, OSM_0}{P_1\, OSM_0 + P_1\, OSM_1}$$

Finally, a Critical Success Index (CSI) was applied. This performance metric accounts for both the over and under prediction of buildings with values ranging from 0 (no match between population and OSM data) and 1 (perfect match between population and OSM data).

$$CSI = \frac{P_1\, OSM_1}{P_1\, OSM_1 + P_0\, OSM_1 + P_1\, OSM_0}$$

The results of the validation exercise outlining the ability of the HRSL, WorldPop and LandScan™ to replicate ~74000 OSM buildings is shown in Tables 3-5. HR scores shown in Table 3 indicate that the vast majority of the buildings defined in the OSM data are also populated in each of the population datasets. An exception to this is the LandScan™ data for the Philippines, where hit rates drop to ~70%. On closer inspection, this drop in HR is a result of the LandScan™ data struggling to replicate coastal populations; at ~1km resolution, the data does not represent the coastline with the same fidelity as the OSM data.

False positive ratios indicate that for both the WorldPop and LandScan™ datasets, the majority of the cells identified as being populated are incorrectly identified as such (Table 4). For example, in Haiti, both datasets return FP scores of 0.9, indicating that 90% of the populated cells are identified as non-building cells in the OSM data. FP scores for HRSL data indicate that most cells identified as being populated are also building cells in the OSM data; a mean FP ratio of ~0.1 indicates that around 10% of the populated cells in the HRSL data are incorrectly identified. The high FP ratios for both the WorldPop and LandScan™ data result in CSI scores being reduced for both datasets; CSI scores averaged across all of the test

regions were 0.88, 0.19 and 0.18 for the HRSL, WorldPop and LandScan™ datasets respectively (Table 5). CSI scores of less than 0.5 indicate that identification of populated cells in, WorldPop and LandScan™ data for these four sites is more wrong than right.

Given the nature of how populations are distributed in both the WorldPop and LandScan™ datasets, the performance metrics shown here are unsurprising, and the results confirm quantitatively what a simple visual inspection immediately shows. Both datasets distribute populations amongst almost all the test area cells, therefore although high HR scores are returned these are mirrored by high FP scores and consequently low CSI values. It is clear that WorldPop and LandScan™ both distribute population counts homogenously (and erroneously) across large areas of lowland floodplain. Both datasets struggle to represent concentrations of exposure, with the total exposed population being spread too thinly over larger areas. HRs for the HRSL are also high, with ~95% of the OSM buildings being represented as populated cells. However, unlike the other population data, the HRSL returned low FP scores and consequently a high CSI of 0.88 across all areas. This result indicates that the majority of the populated cells in the HRSL correspond to building cells in the OSM data, and therefore indicates that the HRSL has considerable skill in replicating buildings. Whilst we cannot at present validate the particular algorithm used to assign enumeration area scale population data to individual buildings, the HRSL data do at least have the potential to be correct at building scales unlike the WorldPop and LandScan™ data which can never achieve this. We can however state that the HRSL data correctly reproduce aggregate census data and identify individual buildings and settlements with high skill. Given that the algorithm makes sensible choices over rules to disaggregate population to these buildings it is likely that the HRSL data also has at least reasonable skill in estimating building level populations whilst this is impossible with other candidate gridded population data sets.

Supplementary References

1. Ward, P. J. *et al.* Assessing flood risk at the global scale: model setup, results, and sensitivity. *Environ. Res. Lett.* **8**, 044019 (2013).
2. Bhaduri, B., Bright, E., Coleman, P. & Urban, M. L. LandScan USA: a high-resolution geospatial and temporal modeling approach for population distribution and dynamics. *GeoJournal* **69**, 103–117 (2007).
3. Hirabayashi, Y. *et al.* Global flood risk under climate change. *Nat. Clim. Change* **3**, 816–821 (2013).
4. Center For International Earth Science Information Network-CIESIN-Columbia University; Centro Internacional De Agricultura Tropical-CIAT. Gridded Population of the World, Version 3 (GPWv3): Population Density Grid. (2005). doi:10.7927/H4XK8CG2
5. Global assessment report on disaster risk reduction 2015 - UNISDR. Available at: https://www.unisdr.org/we/inform/publications/42809. (Accessed: 19th April 2018)
6. Trigg, M. A. *et al.* The credibility challenge for global fluvial flood risk analysis. *Environ. Res. Lett.* **11**, 094014 (2016).
7. Stevens, F. R., Gaughan, A. E., Linard, C. & Tatem, A. J. Disaggregating census data for population mapping using random forests with remotely-sensed and ancillary data. *PloS One* **10**, e0107042 (2015).
8. Kinoshita, Y., Tanoue, M., Watanabe, S. & Hirabayashi, Y. Quantifying the effect of autonomous adaptation to global river flood projections: application to future flood risk assessments. *Environ. Res. Lett.* **13**, 014006 (2018).
9. Klein Goldewijk, K., Beusen, A. & Janssen, P. Long-term dynamic modeling of global population and built-up area in a spatially explicit way: HYDE 3.1. *The Holocene* **20**, 565–573 (2010).
10. Pickard, B. R., Daniel, J., Mehaffey, M., Jackson, L. E. & Neale, A. EnviroAtlas: A new geospatial tool to foster ecosystem services science and resource management. *Ecosyst. Serv.* **14**, 45–55 (2015).
11. Wing, O. E. *et al.* Estimates of present and future flood risk in the conterminous United States. *Environ. Res. Lett.* **13**, 034023 (2018).

12. Horritt, M. . & Bates, P. . Effects of spatial resolution on a raster based model of flood flow. *J. Hydrol.* **253**, 239–249 (2001).

13. Fewtrell, T. J., Bates, P. D., Horritt, M. & Hunter, N. M. Evaluating the effect of scale in flood inundation modelling in urban environments. *Hydrol. Process.* **22**, 5107–5118 (2008).

14. Lwin, K. & Murayama, Y. A GIS Approach to Estimation of Building Population for Micro-spatial Analysis. *Trans. GIS* **13**, 401–414 (2009).

15. Doxsey-Whitfield, E. *et al.* Taking Advantage of the Improved Availability of Census Data: A First Look at the Gridded Population of the World, Version 4. *Pap. Appl. Geogr.* **1**, 226–234 (2015).

16. Tobler, W., Deichmann, U., Gottsegen, J. & Maloy, K. World population in a grid of spherical quadrilaterals. *Int. J. Popul. Geogr.* **3**, 203–225 (1997).

17. Balk, D. L. *et al.* Determining Global Population Distribution: Methods, Applications and Data. in *Advances in Parasitology* **62**, 119–156 (Elsevier, 2006).

18. Deichmann, U., Balk, D. & Yetman, G. Transforming population data for interdisciplinary usages: from census to grid. *Wash. DC Cent. Int. Earth Sci. Inf. Netw.* **200**, (2001).

19. Haklay, M. How Good is Volunteered Geographical Information? A Comparative Study of OpenStreetMap and Ordnance Survey Datasets. *Environ. Plan. B Plan. Des.* **37**, 682–703 (2010).
